# The effect of ageing on the resolution of inflammation

**DOI:** 10.1016/j.arr.2019.101000

**Published:** 2020-01

**Authors:** Wezi Sendama

**Affiliations:** a Translational and Clinical Research Institute, Newcastle University, Newcastle upon Tyne, NE2 4HH, United Kingdom; b The Newcastle upon Tyne Hospitals NHS Foundation Trust, Newcastle upon Tyne, NE7 7DN, United Kingdom

**Keywords:** Ageing, Inflammaging, Resolution of inflammation, Apoptosis, Efferocytosis

## Abstract

Ageing is associated with the development of a low-level, systemic, chronic inflammation known as “inflammaging”. This chronic inflammatory state can contribute to diseases of ageing such as sarcopenia and frailty. The presence of inflammaging suggests a failure of the cell clearance mechanisms that ordinarily aid in the resolution of inflammation after pathogen infiltration or tissue injury. This review aims to explore what is known of how the processes involved in the resolution of inflammation might become defective with age.

## Highlights

• Age-related chronic inflammation (inflammaging) contributes to diseases of ageing.
• Inflammaging suggests age-associated impairment of the resolution of inflammation.
• Human models of inflammation in ageing will be useful in defining these defects.

## 1 Introduction

Acute inflammation is the set of processes that constitute the body’s initial response to tissue injury that might be associated with an invading pathogen. Sterile and non-sterile tissue injury both result in acute inflammation, and the cellular responses that are triggered are geared towards limiting microbial infection and repairing damaged tissues.

**Fig. 1:**
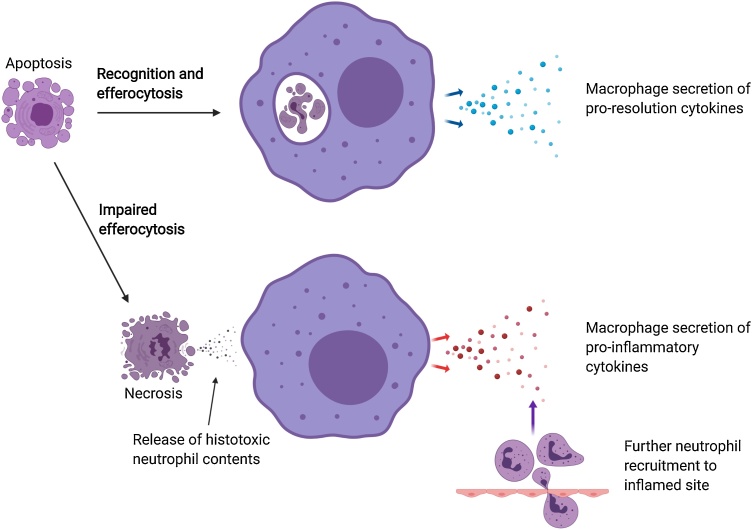
Macrophage efferocytosis. An apoptotic neutrophil at an inflamed site is recognised by a macrophage and phagocytosed. The interaction of the macrophage with the apoptotic cell results in macrophage secretion of pro-resolution cytokines and downregulation of pro-inflammatory cytokines. If efferocytosis is impaired (as in ageing), the apoptotic neutrophil may progress to secondary necrosis, allowing leakage of histotoxic contents, resulting in tissue injury, further pro-inflammatory signalling and chronic inflammation. Image created with BioRender.com.

Inflammation is characterised by infiltration of the injured or invaded site by extravasated neutrophils. Following extravasation, neutrophils and other inflammatory cells can undergo forms of programmed cell death that render them liable to be phagocytosed by macrophages in order to restore tissue homeostasis as inflammation resolves (Fig. 1). This particular form of phagocytosis by macrophages – termed “efferocytosis” – provides a safe means to dispose of apoptotic neutrophils that would otherwise progress to a state of secondary necrosis during which histotoxic neutrophil contents could leak into the local environment and cause tissue damage (Rydell-Törmänen et al., 2006). Efferocytosis also appears to polarise macrophages to a pattern of behaviour that acts to limit the recruitment of further neutrophils as a pathogen is cleared, promoting the resolution of inflammation and restoring tissue homeostasis (Huynh et al., 2002).

It is recognised that with age the mechanisms regulating inflammation become impaired in a manner that might contribute to a susceptibility of older people to infection and age-related chronic diseases (Pawelec et al., 2014). This dysregulation has been inferred in part from an observation of increased circulating pro-inflammatory cytokines and acute phase proteins in older people even in the absence of infection, suggesting a low-level chronic inflammation of ageing known as “inflammaging” (Bartlett et al., 2012; Ferrucci et al., 2005; Pinti et al., 2014). The inappropriate persistence of inflammation with age has been postulated to result in tissue damage which results in further inflammation and more tissue damage, leading to the “cycle of inflammaging” and predisposing to age-related diseases such as atherosclerosis and dementia (Baylis et al., 2013).

The development of a persistent age-related inflammation may suggest the development of defects in the processes relating to the resolution of inflammation including efferocytosis. This review explores the roles of efferocytosis and associated signalling in the resolution of inflammation, aiming to assess the existing evidence regarding changes in these processes with ageing.

## 2 The effect of ageing on apoptosis, efferocytosis and macrophage polarisation

### 2.1 The effect of ageing on neutrophil apoptosis

As inflammation resolves, scavenger cells such as macrophages in tissues attempt to clear superfluous neutrophils that have extravasated to respond to an insult. In experiments involving the co-culture of monocyte-derived macrophages with isolated neutrophils, Savill and colleagues demonstrated that the proportion of neutrophils ingested by the macrophages was directly linked to the proportion of neutrophils that were apoptotic (Savill et al., 1989). It was therefore concluded that apoptosis acts as a flag by which macrophages preferentially recognise redundant neutrophils for clearance, suggesting that the process of apoptosis plays a central role in the resolution of inflammation.

During apoptosis, cells undergo morphological changes that result in condensed nuclei, closely packed organelles and lucent cytoplasmic vacuoles (Kerr et al., 1972). Another crucial structural change in apoptotic cells is the oxidation and relocation of the membrane lipid phosphatidylserine, which moves from the inner plasma membrane leaflet to the outer plasma membrane leaflet. The increased outward exposure of oxidised phosphatidylserine by apoptotic cells is one of the best-described signals by which macrophages recognise them for engulfment in preference to non-apoptotic cells (Fadok et al., 1992; Greenberg et al., 2006).

Ageing does not appear to affect the rate at which unstimulated neutrophils undergo spontaneous apoptosis in culture (Fülöp Jr et al., 1997), although this tells us little about how the cells will behave in conditions of inflammation. When attending inflamed environments after extravasation, it is often the case that neutrophils are exposed to signals to delay apoptosis (Luo and Loison, 2008). Ageing seems to reduce the responsiveness of neutrophils to these anti-apoptotic signals, which include granulocyte-macrophage colony stimulating factor (GM-CSF), lipopolysaccharide (LPS) and IL-2. In experiments using isolated neutrophils from the venous blood of healthy younger (aged 20–25) and older human volunteers (aged 65–85), Fülöp et al. found that although GM-CSF, LPS and IL-2 delayed apoptosis in neutrophils from both sets of volunteers, the anti-apoptotic effects of these compounds on the neutrophils from the older volunteers were weaker (Fülöp Jr et al., 1997).

Neutrophils will also be subject to pro-apoptotic stimuli that are present in inflamed environments, but initiation of apoptosis in this way, at least *via* the Fas receptor on the cell surface that can trigger the extrinsic apoptotic pathway, appears unaffected by age. Tortorella et al. identified similar neutrophil surface expression of Fas in neutrophils from younger (aged 22–32) and older (aged over 65) human volunteers, and ligation of the Fas receptor by monoclonal anti-Fas IgM resulted in similar rates of apoptosis in both sets of neutrophils. Much like in the Fülöp study, however, Tortorella and colleagues observed a reduced sensitivity of Fas-ligated neutrophils to anti-apoptotic factors such as GM-CSF and LPS with age (Tortorella et al., 1998).

There are scant data on the impact of ageing on phosphatidylserine externalisation during apoptosis. Espino and colleagues provide some useful results within a study investigating the modulatory effect of melatonin on leukocyte apoptosis. The findings suggest that neutrophils isolated from the peripheral blood of healthy older human volunteers (aged 65–75) externalised greater amounts of phosphatidylserine than neutrophils from healthy younger volunteers (aged 20–30) when stimulated to undergo apoptosis *in vitro* with the endoplasmic reticulum stressor thapsigargin or the ROS-inducing formyl peptide fMLP (Espino et al., 2011). The results must be accepted with caution as the externalisation of phosphatidylserine was quantified using a fluorophore-conjugated annexin V binding assay, and some authors have expressed reservations about the sensitivity of fluorometric assays for the assessment of absolute numbers of externalised phosphatidylserine molecules rather than the less precise assessment of the loss of membrane lipid asymmetry (Fabisiak et al., 2014; Morita et al., 2011).

### 2.2 The effect of ageing on tissue macrophage chemotaxis

For efferocytosis to take place it is often necessary for macrophages to migrate towards regions of an inflammatory milieu containing greater numbers of apoptotic cells. To promote this, apoptotic cells release chemotactic mediators that serve to draw macrophages towards them. Lauber et al. found that culture supernatants of varieties of cells irradiated to induce apoptosis were able to stimulate migration of macrophages and monocytes. Further testing established one of the chemotactic factors to be the phospholipid lysophosphatidylcholine (LPC), which was released by the apoptotic cells after initiation of apoptosis (Lauber et al., 2003).

As well as lipid chemoattractants such as LPC and sphingosine-1-phosphate, apoptotic cells can secrete nucleotides that attract scavenger cells. The nucleotides ATP and UDP, found in greater concentrations in the supernatants of apoptotic primary thymocytes and Jurkat T cells by Elliott et al., were shown to have monocyte chemoattractant ability *in vitro* and *in vivo* through their interactions with P2Y purinergic receptors on monocytic cells (Elliott et al., 2009).

The effect of ageing on macrophage chemotaxis is poorly investigated, especially where human macrophages are concerned. It might be tempting to draw conclusions about macrophage chemotaxis from studies of circulating monocyte function, but there is compelling evidence that resident tissue macrophages are of distinct lineage to circulating monocytes. Using mouse models, Hashimoto et al. were able to show that circulating monocytes contributed minimally to populations of tissue macrophages in steady state, even during repopulation after cytoablation (Hashimoto et al., 2013). This is probably not the case during inflammation, however. Peripheral blood monocytes have been observed to enter the human lung during experimental inflammation and subsequently adopt gene expression profiles that suggest an ability to regulate the immune response in a similar manner to alveolar macrophages (Jardine et al., 2019). It is not known how long these monocyte-macrophages stay in the tissues after infiltration and whether (and if so, to what extent) they play a role in the clearance of apoptotic cells.

In any case, there is little evidence to suggest peripheral monocyte chemotactic ability is impaired with age. Two studies have suggested no effect of age on general chemotaxis, although in addition to the caveat regarding the use of monocytes as a model for macrophages, it is worth noting that the monocytes were not tested for chemotaxis towards chemoattractants typically released from apoptotic cells as described above (Gardner et al., 1981; Nielsen et al., 1984).

Primary macrophages have been employed to investigate tissue macrophage chemotaxis in ageing but studies show varying results. Forner et al. observed an age-related decline in chemotaxis towards casein in peritoneal macrophages from mice and guinea pigs (Forner et al., 1994). Wustrow et al. noted increasing chemotactic capability with age in peritoneal macrophages from C57BL/6 mice (Wustrow et al., 1982). The chemoattractant in the latter study was serum incubated with a *Salmonella* species to activate complement protein C5. Given that it is possible to impair chemotaxis specifically towards chemoattractants known to be secreted by apoptotic cells and leave general chemotaxis intact (Yang et al., 2005), evidence regarding age-related change in macrophage movement towards efferocytosis-associated chemotactic factors in particular would be welcomed.

### 2.3 The effect of ageing on macrophage recognition and phagocytosis of apoptotic cells

**Fig. 2:**
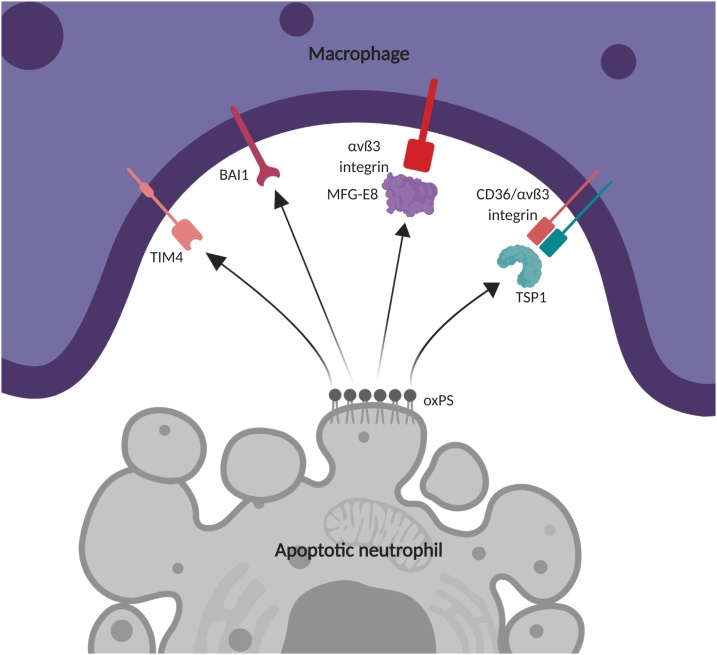
Macrophage recognition of phosphatidylserine on the apoptotic cell surface. Abbreviations: TIM4, T-cell membrane protein 4; BAI1, brain-specific angiogenesis inhibitor 1; MFG-E8, milk fat globule-epidermal growth factor 8; TSP1, thrombospondin 1; oxPS, oxidised phosphatidylserine. Image created with BioRender.com.

Although by no means an exclusive flag for macrophage engulfment, oxidised phosphatidylserine on the apoptotic cell surface can engage with a macrophage to stimulate phagocytosis in a variety of ways (Fig. 2). Some macrophage membrane receptors such as BAI1 and TIM4 have been shown to engage directly with phosphatidylserine, but engagement can also occur *via* bridging molecules such as milk fat globule-epidermal growth factor-factor VIII (MFG-E8), which serves as a link between phosphatidylserine and prophagocytic αvβ3 or αvβ5 integrins on the macrophage surface (Miksa et al., 2007; Poon et al., 2014). Thrombospondin 1 (TSP1) is another bridging molecule that has been shown to tether phosphatidylserine on the surfaces of apoptotic neutrophils to macrophages by binding CD36 and αvβ3 integrin cooperatively on the macrophage surface (Savill et al., 1992). Calreticulin acts in a similar manner after being secreted by macrophages, decorating the surfaces of apoptotic neutrophils to allow engagement by the macrophage prophagocytic receptor CD91. In contrast to MFG-E8 and TSP1, calreticulin adheres to asialoglycans on the target cells rather than externalised phosphatidylserine (Feng et al., 2018).

Despite a lack of interrogation of the mechanisms, it does appear that macrophage phagocytosis of apoptotic cells is diminished with age. In a mouse model, *in vivo* peritoneal macrophages in aged (24-month-old) mice showed impaired efferocytosis of intraperitoneally injected apoptotic Jurkat cells compared to younger (2-month-old) mice (Aprahamian et al., 2008). A similar observation was made by Arnardottir et al. using *in vitro* mouse bone marrow derived macrophages in culture with apoptotic neutrophils isolated from human peripheral blood. Again, the macrophages from aged (20-month-old) mice were less efficient at clearing the neutrophils than macrophages from 2-month-old mice. It was postulated that this contributed to an overall impairment of the resolution of inflammation with increasing age (Arnardottir et al., 2014).

It is unknown whether this defect is mediated by an impaired recognition of apoptotic cells by macrophages, for instance as a consequence of attenuated expression of phosphatidylserine receptors such as TIM4 or reduced production of phosphatidylserine opsonins such as MFG-E8. MFG-E8 knockout mice display features of autoimmunity in a similar pattern to systemic lupus erythematosus, developing elevated serum antinuclear antibodies and glomerulonephritis secondary to renal immunoglobulin deposition (Hanayama et al., 2004). It is suggested that this might be due to impaired efferocytosis of apoptotic B cells or perhaps the impaired maturation of mononuclear cell phagosomes; poor efferosome maturation could result in aberrant cross presentation of apoptotic cell-derived self antigens to T cells (Hanayama et al., 2004; Peng and Elkon, 2011). Although this autoimmune syndrome is not quite consistent with typical observations of age-related chronic neutrophilic inflammation, it is worth noting that some authors have proposed an association between ageing and autoimmunity given that elevated serum antinuclear antibodies can be demonstrated in older, healthy human populations (Andersen-Ranberg et al., 2004; Manoussakis et al., 1987).

By contrast, TIM4 knockout mice display a phenotype that is characterised by an increased cellularity of the peritoneum in homeostasis with elevated concentrations of the pro-inflammatory cytokine TNF-α in peritoneal lavage fluid (Wong et al., 2010). It is probably more enticing to draw parallels between this phenotype and that observed in the ageing human lung, in which increased cellularity and IL-8 concentrations in BAL fluid can be found at steady state (Meyer et al., 1998).

### 2.4 The effect of ageing on macrophage polarisation

It is possible that the features of inflammaging arise due to a failure of macrophages to polarise to a phenotype that favours the resolution of inflammation. A pro-resolution pattern of macrophage behaviour (enhanced efferocytosis, enhanced secretion of anti-inflammatory and pro-resolution cytokines) is often referred to as alternative macrophage activation or M2 activation. It is important to note that while the commonly used nomenclature of “M1″ and “M2″ activation implies a linear spectrum or dichotomy of pro- or anti-inflammatory polarisation, a more accurate model is probably that of multidimensional polarisation in response to variations in the macrophage microenvironment (Byrne et al., 2016).

The mechanisms that govern the change in macrophage behaviour at the time of efferocytosis are not fully understood. The interaction between phosphatidylserine on the outer surface of an apoptotic cell and direct or indirect phosphatidylserine receptors on the macrophage surface does appear to play a role, however, and the alteration in signalling profile looks to take place even prior to target engulfment or internalisation. When actin polymerisation is inhibited with cytochalasin D to limit macrophage engulfment of prey, contact between apoptotic cells and macrophages is still enough to produce a diminution of NF-κB-dependent transcription of genes coding for pro-inflammatory cytokines (Cvetanovic and Ucker, 2004).

It is not clear to what extent the polarisation of macrophages by the efferocytosis of apoptotic material shares the mechanisms of polarisation as a result of other stimuli such as IL-4, IL-10, corticosteroids or specialised pro-resolving mediators. If the mechanisms are similar, then there is potentially relevant evidence that ageing might impair polarisation. In a study comparing mice aged 18–20 months to younger mice aged 10–12 weeks, splenic macrophages from the older mice showed less pronounced mRNA expression of M2-associated genes such as Arg1, FIZZ1 and Ym1 compared to macrophages from the younger mice when pre-stimulated with IL-4 (Mahbub et al., 2012). Bone marrow-derived macrophages differentiated *ex vivo* did not display the same age-associated impairment of polarisation, leading the researchers to conclude that the defect might arise due to an alteration of the tissue microenvironment with ageing rather than some intrinsic change in macrophage function.

#### 2.4.1 Ageing and specialised pro-resolving mediators

Macrophages can be polarised to a pro-resolution phenotype by specialised pro-resolving mediators (SPMs), a superfamily of bioactive substances biosynthesised from essential fatty acids. SPMs include the lipoxins, resolvins, protectins and maresins, which serve to resolve inflammation actively by mechanisms that include enhanced macrophage efferocytosis and the inhibition of further neutrophil extravasation (Serhan, 2014).

In a model employing intraperitoneal zymosan challenge to induce a spontaneously-resolving peritonitis in mice, Arnadottir and colleagues found that an observed delay in resolution of inflammation in older (20 month old) mice compared to younger (2 month old) mice was associated with lower levels of SPMs in peritoneal lavage fluid (Arnardottir et al., 2014). Human monocytes reprogrammed by incubation with SPMs took on a phenotype similar to M2 macrophages and were able to promote resolution when injected into inflamed mouse peritoneal cavities, although the question of whether age affected the extent to which human monocytes could be reprogrammed was not in the scope of the study.

It is possible that ageing impairs the biosynthesis of SPMs. Halade et al. reported similar levels of arachidonic acid-derived eicosanoids (including lipoxin B_4_) in the spleens of young (6 months) and aged 18 months) mice up until the induction of myocardial infarction by coronary artery ligation, at which point splenic levels of eicosanoids decreased to a greater extent in aged mice than young mice. Ageing was associated with reduced mRNA expression of lipoxygenase enzymes, which are essential in the derivation of eicosanoids from arachidonic acid (Halade et al., 2016). The conclusion that ageing might impair production of pro-resolution eicosanoids is shared by Gangemi and colleagues, who found that ageing was associated with reduced urinary lipoxin A_4_ in healthy human volunteers (Gangemi et al., 2005).

## 3 Discussion

The onset of inflammaging suggests a combination of inappropriate initiation of inflammation and inadequate resolution of inflammation with age. The potential contributors to inappropriate pro-inflammatory responses in ageing have been described in detail elsewhere (Franceschi and Campisi, 2014; Singh and Newman, 2011) but the factors that might contribute to defects in the resolution of inflammation are perhaps less well documented.

Mononuclear phagocytes in tissues play an important role in the resolution of inflammation by clearing apoptotic material at inflamed sites and undertaking pro-resolution signalling. Some of the evidence reviewed above from animal models would suggest that the ability of macrophages to recognise and clear apoptotic cells is impaired with age, but if these findings are to lead to therapies to combat inflammaging there is a need for such experiments to be carried out using human models of ageing. Such studies might also consider whether tissue-resident macrophages and monocyte-derived macrophages play distinct roles in the resolution of inflammation.

Although cells other than professional phagocytes have been noted to undertake efferocytosis (Juncadella et al., 2013; Lee et al., 2016), the recognition of macrophages as the foremost effectors of efferocytosis means that it is possible to target therapies to these cells to enhance efferocytosis. 3-hydroxyl-3-methylglutaryl coenzyme A reductase inhibitors (statins) have shown promise in this regard. Lovastatin has been shown to enhance efferocytosis in alveolar macrophages obtained from patients with chronic obstructive pulmonary disease (Morimoto et al., 2006), a condition that is often characterised by chronic lung inflammation. Wootton and colleagues were able to demonstrate enhanced efferocytosis by macrophages from patients taking statins as they recovered from pneumonia (Wootton et al., 2016). Studies specifically designed to investigate inflammaging are required to discern whether such therapies targeted at efferocytosis can blunt the chronic inflammation of ageing and limit the impact of associated tissue damage.

Specialised pro-resolving mediators appear promising in the bid to reverse age-related deficiencies in the resolution of inflammation. As reviewed above, human monocytes reprogrammed by such lipid mediators can enhance resolution of experimental inflammation in the mouse peritoneal cavity (Arnardottir et al., 2014), but it is not yet known whether these substances can be fashioned into drugs that reliably attenuate age-related inflammation in humans. The association of dysregulation of these mediators with the development of age-related diseases such as Alzheimer’s disease (Wang et al., 2015) and atherosclerosis (Laguna-Fernandez et al., 2018) suggests that the pathways could be exploited for patient benefit, but again, further translational studies are required if SPM-modulating therapies to enhance the resolution of inflammaging are to be developed.

## Funding

WS is supported by the Medical Research Council SHIELD antimicrobial resistance consortium (MR/N02995X/1), the Medical Research Foundation National PhD Training Programme in Antimicrobial Resistance Research (MRF-145-0004-TPG-AVISO), and the NIHR Newcastle Biomedical Research Centre (BRC) (IS-BRC-1215-20001). The NIHR Newcastle Biomedical Research Centre (BRC) is a partnership between Newcastle Hospitals NHS Foundation Trust and Newcastle University, funded by the National Institute for Health Research (NIHR). The views expressed are those of the author and not necessarily those of the NIHR or the Department of Health and Social Care.

## Declarations of Competing Interest

None.

## References

[bib0005] Andersen-Ranberg K., Høier-Madsen M., Wiik A., Jeune B., Hegedüs L. (2004). High prevalence of autoantibodies among Danish centenarians. Clin. Exp. Immunol..

[bib0010] Aprahamian T., Takemura Y., Goukassian D., Walsh K. (2008). Ageing is associated with diminished apoptotic cell clearance in vivo. Clin. Exp. Immunol..

[bib0015] Arnardottir H.H., Dalli J., Colas R.A., Shinohara M., Serhan C.N. (2014). Aging delays resolution of acute inflammation in mice: reprogramming the host response with novel nano-proresolving medicines. J. Immunol..

[bib0020] Bartlett D.B., Firth C.M., Phillips A.C., Moss P., Baylis D., Syddall H., Sayer A.A., Cooper C., Lord J.M. (2012). The age-related increase in low-grade systemic inflammation (Inflammaging) is not driven by cytomegalovirus infection. Aging Cell.

[bib0025] Baylis D., Bartlett D.B., Patel H.P., Roberts H.C. (2013). Understanding how we age: insights into inflammaging. Longev. Heal..

[bib0030] Byrne A.J., Maher T.M., Lloyd C.M. (2016). Pulmonary Macrophages: A New Therapeutic Pathway in Fibrosing Lung Disease?. Trends Mol. Med..

[bib0035] Cvetanovic M., Ucker D.S. (2004). Innate immune discrimination of apoptotic cells: repression of proinflammatory macrophage transcription is coupled directly to specific recognition. J. Immunol..

[bib0040] Elliott M.R., Chekeni F.B., Trampont P.C., Lazarowski E.R., Kadl A., Walk S.F., Park D., Woodson R.I., Ostankovich M., Sharma P., Lysiak J.J., Harden T.K., Leitinger N., Ravichandran K.S. (2009). Nucleotides released by apoptotic cells act as a find-me signal to promote phagocytic clearance. Nature.

[bib0045] Espino J., Bejarano I., Paredes S.D., Barriga C., Reiter R.J., Pariente J.A., Rodríguez A.B. (2011). Melatonin is able to delay endoplasmic reticulum stress-induced apoptosis in leukocytes from elderly humans. Age (Omaha)..

[bib0050] Fabisiak J.P., Borisenko G.G., Kagan V.E. (2014). Quantitative method of measuring phosphatidylserine externalization during apoptosis using electron paramagnetic resonance (EPR) spectroscopy and annexin-conjugated iron. Methods Mol. Biol..

[bib0055] Fadok V.A., Voelker D.R., Campbell P.A., Cohen J.J., Bratton D.L., Henson P.M. (1992). Exposure of phosphatidylserine on the surface of apoptotic lymphocytes triggers specific recognition and removal by macrophages. J. Immunol..

[bib0060] Feng M., Marjon K.D., Zhu F., Weissman-Tsukamoto R., Levett A., Sullivan K., Kao K.S., Markovic M., Bump P.A., Jackson H.M., Choi T.S., Chen J., Banuelos A.M., Liu J., Gip P., Cheng L., Wang D., Weissman I.L. (2018). Programmed cell removal by calreticulin in tissue homeostasis and cancer. Nat. Commun..

[bib0065] Ferrucci L., Corsi A., Lauretani F., Bandinelli S., Bartali B., Taub D.D., Guralnik J.M., Longo D.L. (2005). The origins of age-related proinflammatory state. Blood.

[bib0070] Forner M.A., Collazos M.E., Barriga C., De la Fuente M., Rodriguez A.B., Ortega E. (1994). Effect of age on adherence and chemotaxis capacities of peritoneal macrophages. Influence of physical activity stress. Mech. Ageing Dev..

[bib0075] Franceschi C., Campisi J. (2014). Chronic inflammation (Inflammaging) and its potential contribution to age-associated diseases. Journals Gerontol. - Ser. A Biol. Sci. Med. Sci..

[bib0080] Fülöp T., Fouquet C., Allaire P., Perrin N., Lacombe G., Stankova J., Rola-Pleszczynski M., Gagné D., Wagner J., Khalil A., Dupuis G. (1997). Changes in apoptosis of human polymorphonuclear granulocytes with aging. Mech. Ageing Dev..

[bib0085] Gangemi S., Pescara L., D’Urbano E., Basile G., Nicita-Mauro V., Davì G., Romano M. (2005). Aging is characterized by a profound reduction in anti-inflammatory lipoxin A4 levels. Exp. Gerontol..

[bib0090] Gardner I.D., Lim S.T.K., Lawton J.W.M. (1981). Monocyte function in ageing humans. Mech. Ageing Dev..

[bib0095] Greenberg M.E., Sun M., Zhang R., Febbraio M., Silverstein R., Hazen S.L. (2006). Oxidized phosphatidylserine-CD36 interactions play an essential role in macrophage-dependent phagocytosis of apoptotic cells. J. Exp. Med..

[bib0100] Halade G.V., Kain V., Black L.M., Prabhu S.D., Ingle K.A. (2016). Aging dysregulates D- and E-series resolvins to modulate cardiosplenic and cardiorenal network following myocardial infarction. Aging (Albany. NY)..

[bib0105] Hanayama R., Tanaka M., Miyasaka K., Aozasa K., Koike M., Uchiyama Y., Nagata S. (2004). Autoimmune disease and impaired uptake of apoptotic cells in MFG-E8-deficient mice. Science.

[bib0110] Hashimoto D., Chow A., Noizat C., Teo P., Beasley M.B., Leboeuf M., Becker C.D., See P., Price J., Lucas D., Greter M., Mortha A., Boyer S.W., Forsberg E.C., Tanaka M., van Rooijen N., García-Sastre A., Stanley E.R., Ginhoux F., Frenette P.S., Merad M. (2013). Tissue-resident macrophages self-maintain locally throughout adult life with minimal contribution from circulating monocytes. Immunity.

[bib0115] Huynh M.-L.N., Fadok V.A., Henson P.M. (2002). Phosphatidylserine-dependent ingestion of apoptotic cells promotes TGF-beta1 secretion and the resolution of inflammation. J. Clin. Invest..

[bib0120] Jardine L., Wiscombe S., Reynolds G., McDonald D., Fuller A., Green K., Filby A., Forrest I., Ruchaud-Sparagano M.-H., Scott J., Collin M., Haniffa M., Simpson A.J. (2019). Lipopolysaccharide inhalation recruits monocytes and dendritic cell subsets to the alveolar airspace. Nat. Commun..

[bib0125] Juncadella I.J., Kadl A., Sharma A.K., Shim Y.M., Hochreiter-Hufford A., Borish L., Ravichandran K.S. (2013). Apoptotic cell clearance by bronchial epithelial cells critically influences airway inflammation. Nature.

[bib0130] Kerr J.F., Wyllie A.H., Currie A.R. (1972). Apoptosis: a basic biological phenomenon with wide-ranging implications in tissue kinetics. Br. J. Cancer.

[bib0135] Laguna-Fernandez A., Checa A., Carracedo M., Artiach G., Petri M.H., Baumgartner R., Forteza M.J., Jiang X., Andonova T., Walker M.E., Dalli J., Arnardottir H., Gistera A., Thul S., Wheelock C.E., Paulsson-Berne G., Ketelhuth D.F.J., Hansson G.K., Bäck M. (2018). ERV1/ChemR23 signaling protects against atherosclerosis by modifying oxidized low-density lipoprotein uptake and phagocytosis in macrophages. Circulation.

[bib0140] Lauber K., Bohn E., Kröber S.M., Xiao Y.J., Blumenthal S.G., Lindemann R.K., Marini P., Wiedig C., Zobywalski A., Baksh S., Xu Y., Autenrieth I.B., Schulze-Osthoff K., Belka C., Stuhler G., Wesselborg S. (2003). Apoptotic cells induce migration of phagocytes via caspase-3-mediated release of a lipid attraction signal. Cell.

[bib0145] Lee C.S., Penberthy K.K., Wheeler K.M., Juncadella I.J., Vandenabeele P., Lysiak J.J., Ravichandran K.S. (2016). Boosting apoptotic cell clearance by colonic epithelial cells attenuates inflammation in vivo. Immunity.

[bib0150] Luo H.R., Loison F. (2008). Constitutive neutrophil apoptosis: mechanisms and regulation. Am. J. Hematol..

[bib0155] Mahbub S., Deburghgraeve C.R., Kovacs E.J. (2012). Advanced age impairs macrophage polarization. J. Interferon Cytokine Res..

[bib0160] Manoussakis M.N., Tzioufas A.G., Silis M.P., Pange P.J., Goudevenos J., Moutsopoulos H.M. (1987). High prevalence of anti-cardiolipin and other autoantibodies in a healthy elderly population. Clin. Exp. Immunol..

[bib0165] Meyer K.C., Rosenthal N.S., Soergel P., Peterson K. (1998). Neutrophils and low-grade inflammation in the seemingly normal aging human lung. Mech. Ageing Dev..

[bib0170] Miksa M., Amin D., Wu R., Ravikumar T.S., Wang P., Wang P. (2007). Fractalkine-induced MFG-E8 leads to enhanced apoptotic cell clearance by macrophages. Mol. Med..

[bib0175] Morimoto K., Janssen W.J., Fessler M.B., McPhillips K.A., Borges V.M., Bowler R.P., Xiao Y.-Q., Kench J.A., Henson P.M., Vandivier R.W. (2006). Lovastatin enhances clearance of apoptotic cells (Efferocytosis) with implications for chronic obstructive pulmonary disease. J. Immunol..

[bib0180] Morita S., Shirakawa S., Kobayashi Y., Nakamura K., Teraoka R., Kitagawa S., Terada T. (2011). Enzymatic measurement of phosphatidylserine in cultured cells. J. Lipid Res..

[bib0185] Nielsen H., Blom J., Larsen S.O. (1984). Human blood monocyte function in relation to age. Acta Pathol. Microbiol. Immunol. Scand. C..

[bib0190] Pawelec G., Goldeck D., Derhovanessian E. (2014). Inflammation, ageing and chronic disease. Curr. Opin. Immunol..

[bib0195] Peng Y., Elkon K.B. (2011). Autoimmunity in MFG-E8-deficient mice is associated with altered trafficking and enhanced cross-presentation of apoptotic cell antigens. J. Clin. Invest..

[bib0200] Pinti M., Cevenini E., Nasi M., De Biasi S., Salvioli S., Monti D., Benatti S., Gibellini L., Cotichini R., Stazi M.A., Trenti T., Franceschi C., Cossarizza A. (2014). Circulating mitochondrial DNA increases with age and is a familiar trait: Implications for inflamm-aging. Eur. J. Immunol..

[bib0205] Poon I.K.H., Lucas C.D., Rossi A.G., Ravichandran K.S. (2014). Apoptotic cell clearance: basic biology and therapeutic potential. Nat. Rev. Immunol..

[bib0210] Rydell-Törmänen K., Uller L., Erjefält J.S. (2006). Direct evidence of secondary necrosis of neutrophils during intense lung inflammation. Eur. Respir. J..

[bib0215] Savill J., Hogg N., Ren Y., Haslett C. (1992). Thrombospondin cooperates with CD36 and the vitronectin receptor in macrophage recognition of neutrophils undergoing apoptosis. J. Clin. Invest..

[bib0220] Savill J.S., Wyllie A.H., Henson J.E., Walport M.J., Henson P.M., Haslett C. (1989). Macrophage phagocytosis of aging neutrophils in inflammation. Programmed cell death in the neutrophil leads to its recognition by macrophages. J. Clin. Invest..

[bib0225] Serhan C.N. (2014). Pro-resolving lipid mediators are leads for resolution physiology. Nature.

[bib0230] Singh T., Newman A.B. (2011). Inflammatory markers in population studies of aging. Ageing Res. Rev..

[bib0235] Tortorella C., Piazzolla G., Spaccavento F., Pece S., Jirillo E., Antonaci S. (1998). Spontaneous and Fas-induced apoptotic cell death in aged neutrophils. J. Clin. Immunol..

[bib0240] Wang X., Zhu M., Hjorth E., Cortés-Toro V., Eyjolfsdottir H., Graff C., Nennesmo I., Palmblad J., Eriksdotter M., Sambamurti K., Fitzgerald J.M., Serhan C.N., Granholm A.C., Schultzberg M. (2015). Resolution of inflammation is altered in Alzheimer’s disease. Alzheimer’s Dement..

[bib0245] Wong K., Valdez P.A., Tan C., Yeh S., Hongo J.-A., Ouyang W. (2010). Phosphatidylserine receptor Tim-4 is essential for the maintenance of the homeostatic state of resident peritoneal macrophages. Proc. Natl. Acad. Sci. U. S. A..

[bib0250] Wootton D.G., Diggle P.J., Court J., Eneje O., Keogan L., Macfarlane L., Wilks S., Woodhead M., Gordon S.B. (2016). Recovery from pneumonia requires efferocytosis which is impaired in smokers and those with low body mass index and enhanced by statins. Thorax.

[bib0255] Wustrow T.P.U., Denny T.N., Fernandes G., Good R.A. (1982). Changes in macrophages and their functions with aging in C57BL/6J, AKR/J, and SJL/J mice. Cell. Immunol..

[bib0260] Yang L.V., Radu C.G., Wang L., Riedinger M., Witte O.N. (2005). Gi-independent macrophage chemotaxis to lysophosphatidylcholine via the immunoregulatory GPCR G2A. Blood.

